# The Effect of Long-Running Severe Selenium-Deficiency on the Amount of Iron and Zinc in the Organs of Rats

**DOI:** 10.3390/molecules14114440

**Published:** 2009-11-05

**Authors:** Ken-ichiro Matsumoto, Sachiyo Terada, Marie Ariyoshi, Aya Okajo, Atsushi Hisamatsu, Iori Ui, Kazutoyo Endo

**Affiliations:** 1 Department of Physical Chemistry, Showa Pharmaceutical University, 3-3165 Higashi-Tamagawagakuen, Machida-shi, Tokyo 194-8543, Japan; E-Mail: matsumok@nirs.go.jp (K.M.); 2 Heavy-Ion Radiobiology Research Group, Research Center for Charged Particle Therapy, National Institute of Radiological Sciences, 4-9-1 Anagawa, Inage-ku, Chiba-shi, Chiba 263-8555, Japan

**Keywords:** instrumental neutron activation analysis, bio-trace element, mineral, aging, oxidative stress

## Abstract

The amounts of selenium (Se), iron (Fe), and zinc (Zn) in the liver, kidney, and spleen as a function of age of rats measured using instrumental neutron activation analysis were compared between Se-deficient (SeD) rats and normal rats. The SeD model rats can live for more than 50 weeks. The effect of Se-deficinecy in rats might be weak, compared to the marked malfunction of GSH-Px. The SeD rats can be considered as a model of non-lethal chronic oxidative stress. Fluctuations of Fe and Zn in the liver of Se-deficient rats were observed. The amount of redox-relating minerals, such as Fe and Zn, in SeD rat organs is changeable depending on the age.

## 1. Introduction

Selenium (Se) is an essential trace element and has important roles in the redox regulation system in our body. Oxidative stress caused by Se deficiency has been studied using a Se-deficient (SeD) rat model, which involves feeding the pregnant mother a torula yeast base SeD diet [1,2]. Se-deficiency results in the depression of glutathione peroxidase (GSH-Px) activity and is believed to cause various forms of oxidative stress [3]; therefore roles and/or amount of biometals working in the *in vivo* redox regulation system, such as iron (Fe) and zinc (Zn), may modify oxidative stresses.

Fe accumulations in several organs of the SeD rat have been reported [1,2,4,5,6]; however, the Fe levels appear to be dependent on the age of the SeD rat [6]. The mechanisms of Fe accumulation and function of Fe accumulated in organs are still under investigation. On the other hand, levels of Zn in several tissues of the SeD rat appear to be decreased compared with normal rats [1]. Zn is necessary for various physiological functions in living organisms and influences the activity of over 300 enzymes. Tissue levels of Zn may also depend on age [7].

In this paper, the dependence of rat age on Se, Fe, and Zn levels in the liver, kidney, and spleen were measured and compared using instrumental neutron activation analysis (INAA). The effect of age-dependent oxidative stress on mineral balances in rat tissues/organs was discussed.

## 2. Results and Discussion

Our SeD model rats lived for more than 50 weeks until being sacrificed at the time of the experimentation. No tumor/cancer and/or no abnormal changes were observed in the internal organs of the SeD rats used in this experiment. The major visible abnormalities of our SeD model rat were ateliosis, alopecia, and aggressiveness, which are also found in the deficiency of other minerals and are not specific to Se-deficiency. The ateliosis and alopecia subsided after 20 weeks of age.

**Figure 1:**
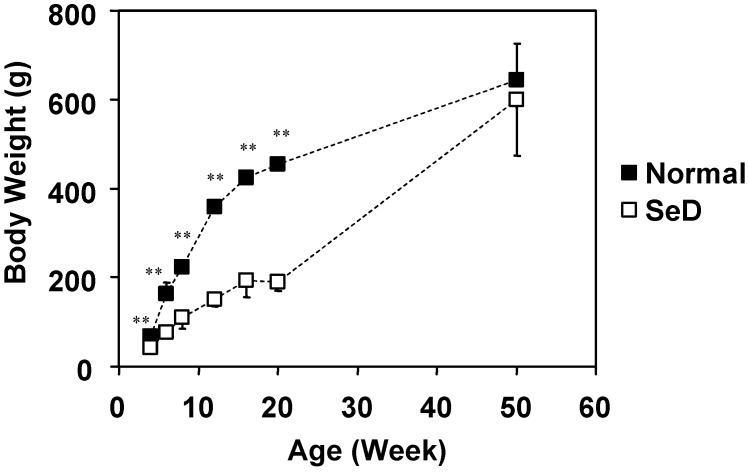
Dependence of body weight of normal and SeD rats on the age of the rats. The values are indicated as the mean ± SD. Significant difference between the normal and SeD groups are indicated by ^**^ at p < 0.001.

The growth curves for body weight of both normal and SeD rat groups are shown in Figure 1. Numbers of rats in 4-, 6-, 8-, 12-, 16-, 20-, and 50-week-old normal rat groups were 10, 32, 37, 16, 28, 10, and 7, respectively. Numbers of rats in 4-, 6-, 8-, 12-, 16-, 20-, and 50-week-old SeD rat groups were 24, 32, 35, 13, 25, 15, and 3, respectively. Data include rats used not only for this paper but also for other experiments in our laboratory. The body weight of the SeD rat was significantly smaller (p < 0.001) than that of normal rats at the same age, except for the 50-week-old group, where no significant difference was observed. The body weight of normal rats followed a logarithmic rate of growth up to 50 weeks of age. On the other hand, the growth of the SeD rat appeared to stagnate at 16–20 weeks of age, but again increased after 20 weeks of age to reach the same body weight level as normal rats at 50 weeks of age.

**Figure 2:**
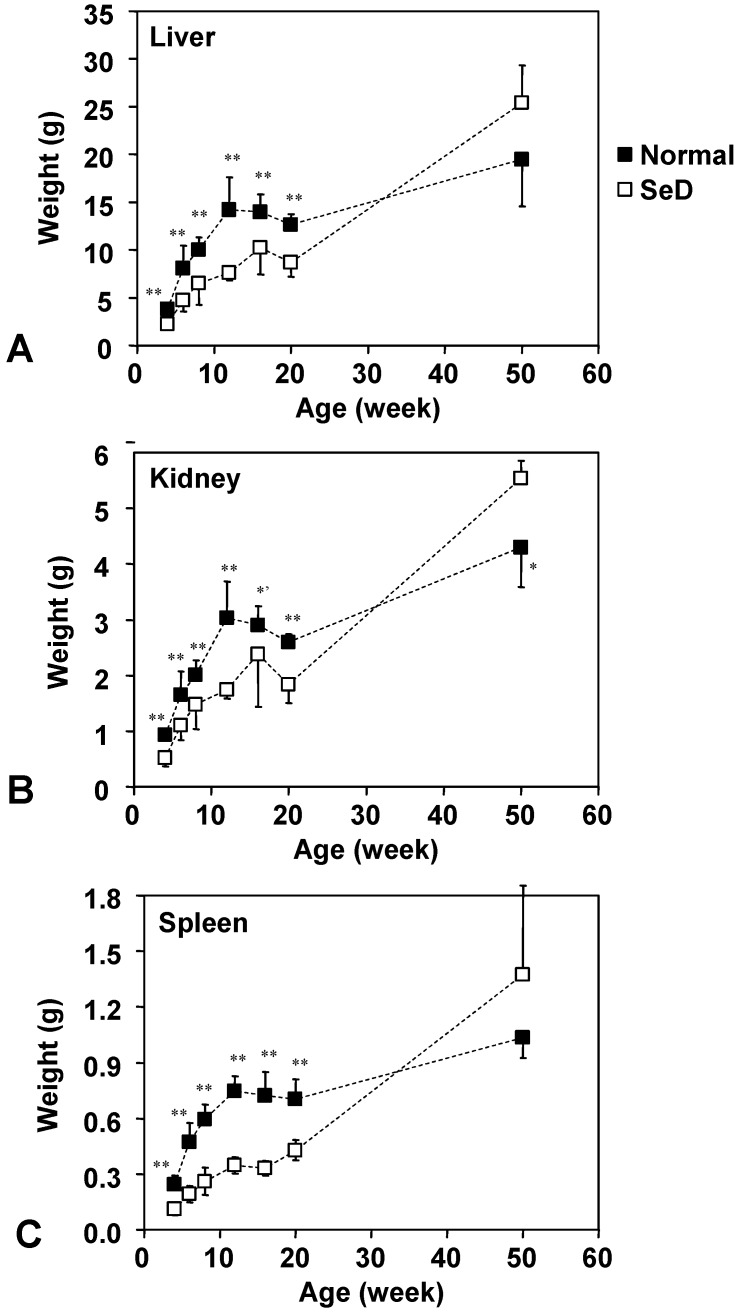
Dependence of weights of the lever, kidney, and spleen of normal and SeD rats on the age of rats. Values are indicated as the mean ± SD. ^*^, ^*’^, and ^**^ indicate significance between normal and SeD groups at p < 0.05, p < 0.01, and p < 0.001, respectively.

The age dependence of the weight of the liver, kidney and spleen is shown in Figure 2. Numbers of rats were the same as in Figure 1. The organ weights of the SeD groups were significantly smaller than those of the normal groups, up until 20 weeks of age; however, no remarkable difference was obtained at 50 weeks of age except that the kidneys of 50-week-old SeD rats were significantly larger than that of the normal group (at p < 0.05). The growth curve of the liver, kidney and spleen of both normal and SeD rats reached a plateau at 12 or 16 weeks of age, but then grew again beyond 20 weeks of age; however, the organ weight of SeD rats increased rapidly following 20 weeks of age to exceed the organ weights of normal rats after 50 weeks of age. It is interesting to note that the profiles of growth curves of organs follow a similar trend to those of the SeD rat body weight (Figure 1).

**Figure 3:**
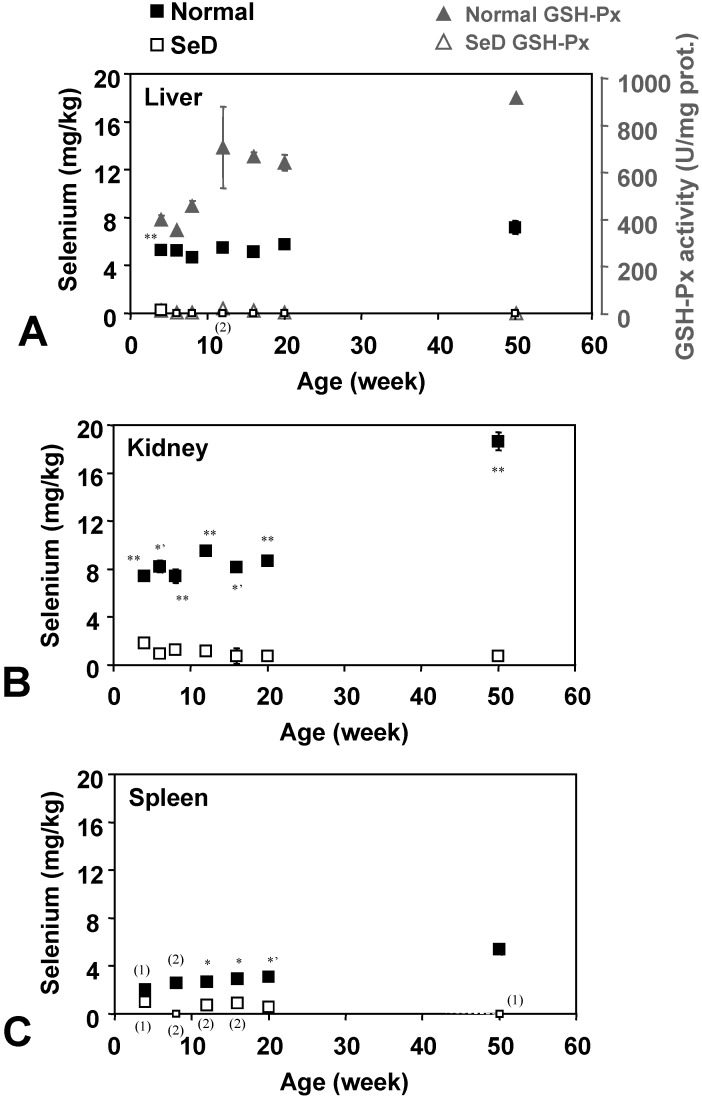
Dependence of Se contents in the (A) liver, (B) kidney, and (C) spleen of normal and SeD rats on the age of rats. Values are indicated as the mean ± SD of three samples. Numbers in parentheses indicate the number of samples when number of samples measured were less than 3. ^*^, ^*’^, and ^**^ indicate significant difference between normal and SeD groups at p < 0.05, p < 0.01, and p < 0.001, respectively. Gray closed and open triangles in (A) indicate GSH-Px activity in normal and SeD rat liver, respectively. Values are indicated as the mean ± SD of 3 samples. GSH-Px activities of SeD groups were significantly lower than that of normalgroup by p < 0.001 for all ages.

Figure 3 shows the age dependence of Se contents in several organs. Small open squares indicate that the level of Se was below the detection limit. Se content was almost below the detection limit in the liver of SeD rats, except for a slight amount (0.27 ± 0.19 mg/kg, n = 3) which was detected in 4-week-old SeD rats; however, Se could be detected in the kidney of all SeD rats tested, although the Se level was close to the detection limit. In the spleen of the SeD group, Se could also be detected at very low levels in the 4-, 12-, 16-, and 20-week-old groups, but not in the 8- and 50-week-old groups. Se contents in normal rat organs were considerably higher than SeD rats of the same age. Se contents of the normal rat increased with age in all three organs tested, although the rate of increase depended on the particular organ tested. Although the tendency of time course of Se contents in normal rat organs is similar among organs, the Se contents are different depending on organs. Markedly higher values were obtained in 50-week-old normal rat kidneys. The age dependence of GSH-Px activities in the liver (Figure 3A, gray triangles) seems to be in sync with Se contents in the liver. GSH-Px activities in SeD rats were effectively absent throughout their life.

**Figure 4:**
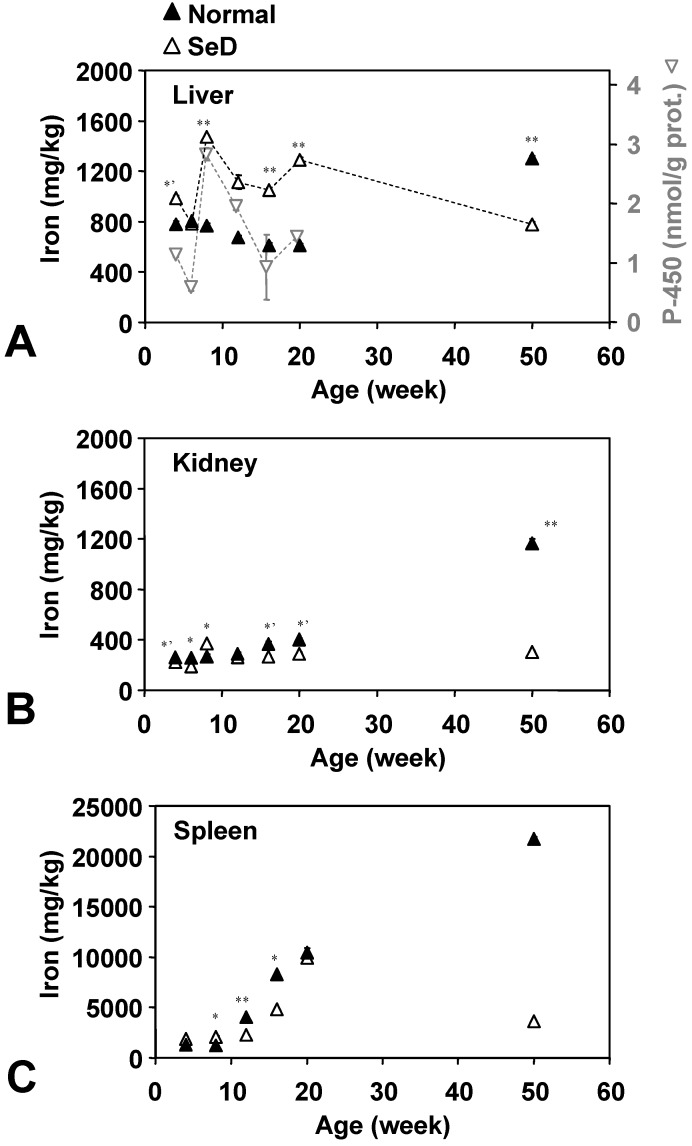
Dependence of Fe contents in the (A) liver, (B) kidney, and (C) spleen of normal and SeD rats on the age of rats. Values are indicated as the mean ± SD of three measurements. ^*^, ^*’^, and ^**^ indicate a significant difference between normal and SeD groups at p < 0.05, p < 0.01, and p < 0.001, respectively. Gray inverse triangles in (A) indicate cytochrome P-450 activity in the SeD rat liver microsome. Values are indicated as the mean ± SD of 3 measurements. Cytochrome P-450 activity of 50-week-old groups was not measured.

The age dependence of Fe levels in several organs is shown in Figure 4. Fe in the normal rat liver gradually decreased from 800 mg/kg at four weeks of age to 600 mg/kg at 20 weeks of age; however, 50-week-old normal rats showed 1,300 mg/kg of Fe in the liver. In younger groups below 20 weeks of age, Fe contents in the SeD rat liver were higher than in normal rats in the same age group, except that Fe levels in 6-week-old SeD rats were similar to the Fe level in normal rats. Fifty-week-old SeD rats showed a lower Fe level than the normal group at the same age. Fe in the SeD rat liver has large age-dependent variation (780–1,500 mg/kg) with an average value of approximately 1,000 mg/kg over the life of the rat. Fe levels in the SeD rat kidney were relatively stable around 190–370 mg/kg, while Fe in the normal rat kidney gradually increased from 260 mg/kg at four weeks of age to 1,160 mg/kg in the 50-week-old group. Fe in the spleen was 1–2 orders higher than the other two organs. Fe in both the SeD and normal rat spleen showed a similar profile up until 20 weeks of age. Fe in the spleen was relatively low until eight weeks of age and then began to increase. In normal rats, Fe in the spleen increased until 50 weeks of age, while it decreased after 20 weeks of age in SeD rats. The 50-week-old SeD rats showed markedly lower Fe contents in the spleen than normal rats. The variation of SeD rat cytochrome P-450 activity (Figure 4A, tray triangles) seems to be in sync with the iron contents of the SeD rat liver. The age dependence of cytochrome P-450 activity in the normal rat liver, however, seems unrelated to Fe contents (data not shown).

**Figure 5:**
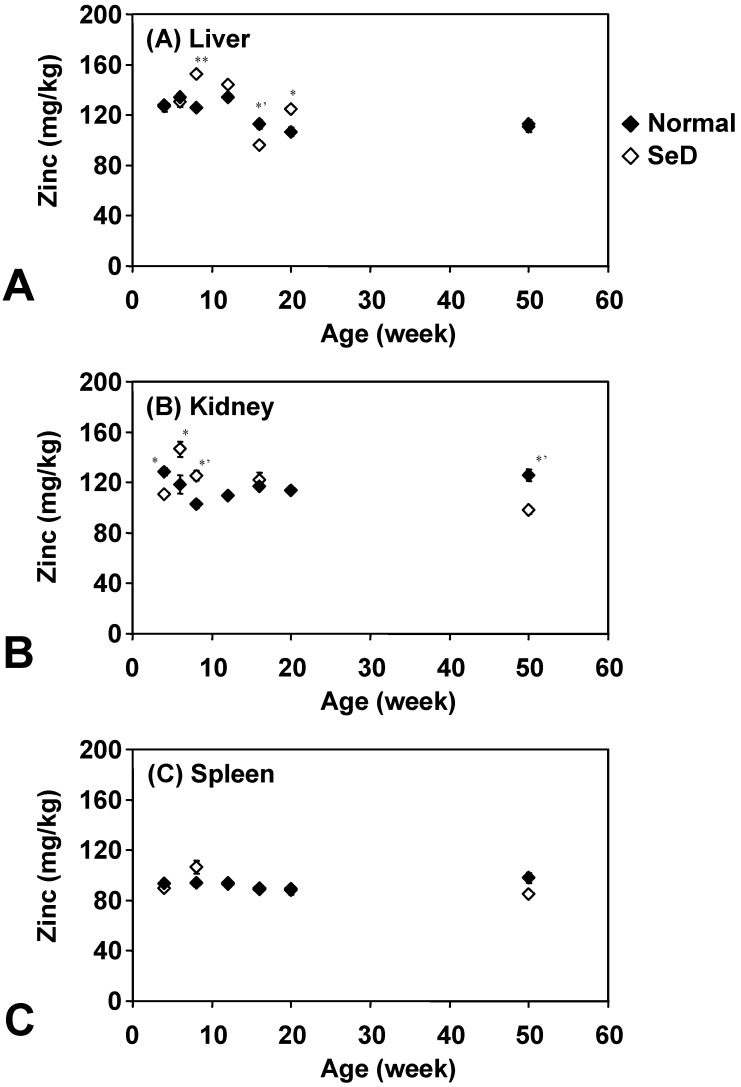
Dependence of Zn contents in the (A) liver, (B) kidney, and (C) spleen of normal and SeD rats on the age of rats. Values are indicated as the mean ±SD of three measurements. ^*^, ^*’^, and ^**^ indicate significant difference between normal and SeD groups at p < 0.05, p < 0.01, and p < 0.001, respectively.

Figure 5 shows the age dependence of Zn levels in several organs. There was no remarkable difference between normal and SeD rats, and also among organs of both rat groups. In addition, Zn in organs was quite stable throughout their life; however, slight variations were obtained in the liver and kidney of younger SeD rats. Zn contents in the normal rat spleen were effectively constant throughout their life.

**Figure 6:**
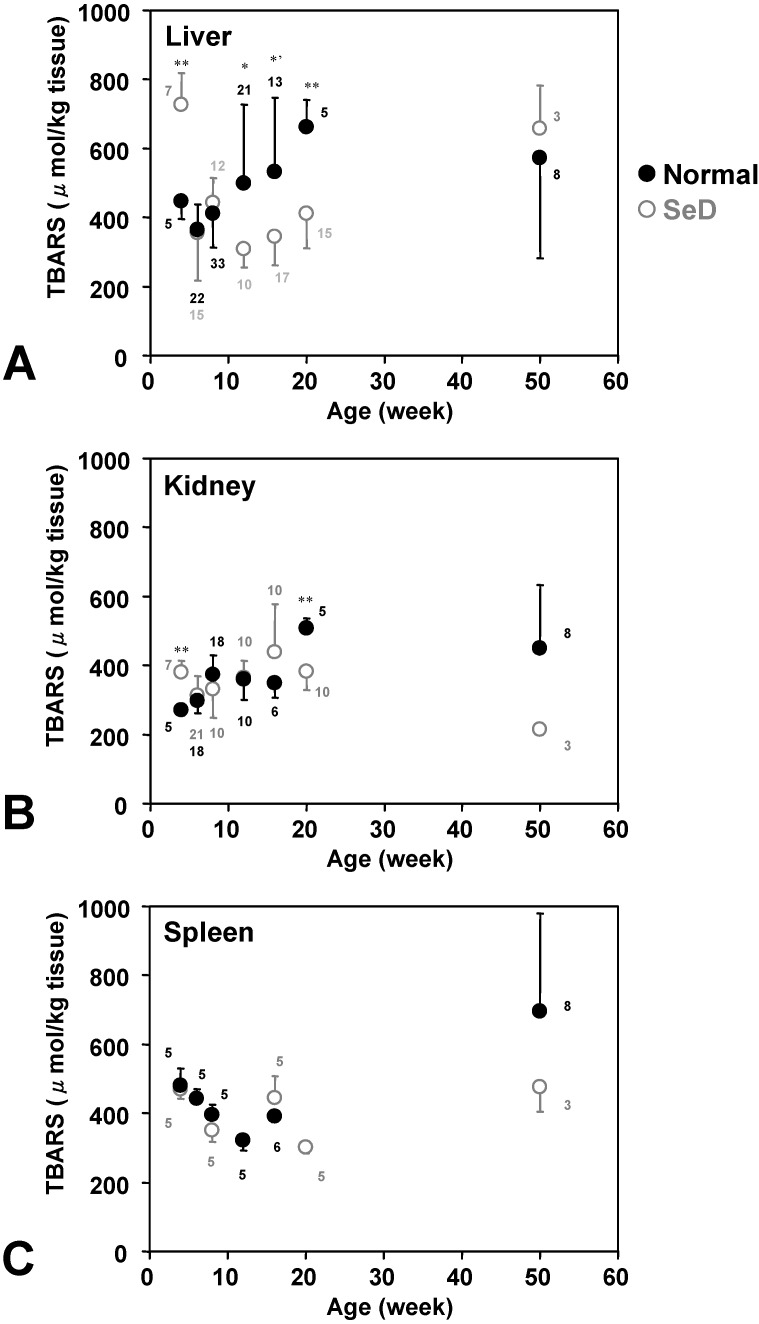
Dependence of TBARS levels in (A) liver, (B) kidney, and (C) spleen of normal and SeD rats on the age of rats. Gray open and black closed circles were indicated values of SeD and normal groups. Values are indicated as the mean ± SD. Numbers indicated in the figures are the number of samples measured. ^*^, ^*’^, and ^**^ indicate significant differences between normal and SeD groups at p < 0.05, p < 0.01, and p < 0.001, respectively.

Figure 6 shows the age dependence of TBARS levels in several organs. TBARS in the liver and kidney of the normal rat gradually increased with age and reached plateau level; however, TBARS in the spleen of normal rats decreased when they were young, was lowest at 12 weeks of age, and then increased again until 50 weeks. The time course of TBARS in the liver of SeD rats showed a similar pattern as in normal rat spleen. The liver and kidney of normal rats showed a similar time-course pattern of TBARS. TBARS values in the kidney and spleen of SeD rats at 50 weeks of age appeared lower than those of normal rats but there was no significance.

Young Se-deficient rats showed marked alopecia and ateliosis, which may be worse around eight weeks of age. An appearance of the rats was, however, recovered to be normal gradually with age after eight weeks old. In this experiment, randomly selected three of three Se-deficient rats could live until 50 weeks old. An appearance of 50 week old Se-deficient rat was almost normal, while their Se contents kept very low level. Se-deficient may give relatively large effects for young generation of rat, but not lethal effects. Se-deficient may give weak chronic stress for elder generation of rat.

The growth rates of normal rat body weight were always higher than the growth rates of organs, when plotted by age, i.e., the percentage difference of body or organ weight per week (data not shown); however, the growth rates of SeD rat body weight were similar to the organs. The growth rates of the organs of both rats and the body weight of the SeD rat had a negative value at 12–20 weeks of age, while the body weight growth rate of normal rats did not have a negative value. This suggested that the increasing body weight of normal rats was dependent not only on substantial organ/tissue weight, but also extra fat, etc. In contrast, increasing body weight of SeD rats was mainly dependent on substantial tissue weight. In fact, less fat can be seen in the SeD rat abdomen than in normal rats at the same age. The growth rates of body and organ weights of normal rats between 20 and 50 weeks ranged from 1.4–2.2% increase/week, and those of SeD rat ranged from 6.4–7.4% increase/week. The increasing body weight of SeD rats from 20–50 weeks was mainly due to the increasing weight of substantial tissues, since the growth rates of SeD rat organs were three times larger in this period.

That recovery of the growth rate of SeD rats might be due to less oxidative stress, because exercise might be restricted with increasing body weight. The motion activity of experimental animals might fall with age, and oxidative stress due to motion might not be present in a large and overweight experimental rat beyond 20 weeks of age. Under such a condition of a limited amount of exercise, Se deficiency can not be lethal, because the animals may not have been exposed to oxidative stress with the explosion of H_2_O_2_ generation. On the other hand, Se deficiency may be more severe in field or cattle animals, which are exposed to chronic oxidative stress due to the minimum required exercise.

The ratio of organ weight to the body weight, however, is larger in Se-deficient rats compared to the normal rats. Organs of Se-deficient rats may have been swollen due to the chronic weak oxidative stress. Such a tendency was also obtained in our previous paper using eight week old rats [1,11].

It has been reported that Se-deficiency leads to white muscle disease, an acute myodegeneration of domestic animals such as calves and lambs [12]. Se-deficiency is also associated with human disorders including Keshan disease and Kaschin-Beck disease [13]. However, Se-deficiency does not cause such disorders in experimental animals. In addition, no clear evidence of therapeutic benefits of Se supplementation was obtained in environmental health disorders [14]. Therefore, it has been predicted that some secondary processes cause those disorders [15]. The stress caused by Se-deficiency itself was not lethal for the animals. The resulting oxidative injury in SeD rats might be weak, compared to the marked malfunction of GSH-Px.

Ho *et al*. reported that the GSH-Px knockout mouse has no increased sensitivity to hyperoxia [16]. GSH-Px may prevent disorders associated with oxidative stress in healthy animals by interaction with other antioxidative enzymes and/or antioxidants. Therefore, malfunction of GSH-Px alone may not be lethal for experimental animals. Nevertheless, the Se-deficiency model can be considered to be a model for “very weak chronic oxidative stress”.

Se and Fe accumulated in older normal rat organs (Figure 3, Figure 4). Accumulation of these minerals may induce another redox state and may cause oxidative stress at the molecular level. The higher TBARS levels caused by such accumulation of Se and Fe in older normal rats had been predicted, while their TBARS levels were stable or somewhat decreased compared with those of the 20-week-old group. The TBARS levels in SeD rat organs seemed roughly inverse to the Fe levels in the corresponding organ. The accumulation of Fe in rat organs can be considered to reduce oxidative stress mediated by free Fe ions. Less motor activity at a higher age may also keep TBARS levels relatively low.

Since similarity between SeD and vitamin E deficient rats to the changes in Fe and Zn contents in several organs were shown in the previous report [2], uptake of redox-related minerals, including Zn, into the tissue may change as a response to oxidative stress. Relatively larger fluctuations of Fe and Zn in the liver of younger SeD rats can be considered as a sensitive response to oxidative stress caused by motor activity of the young rats. The exercise-associated oxidative stress can be severe under the Se-deficiency, but it is mild or weak in the normal rats. The SeD model older rats were not exposed to much oxidative stress because of less motor activity. In other words, young SeD rats are stressed by motor activity. Fe and/or Zn contents in the tissue may be transiently modified by response to oxidative stress.

In this paper, 4-, 8-, 12-, 16-, and 20-week-old SeD rats showed higher Fe contents in the liver than normal rats. Eight-week-old SeD rat also showed higher Fe contents in the kidney compared to normal rats of the same age. In the spleen, slightly higher Fe contents of SeD rats than normal rats were seen below eight weeks of age. One paper showed Fe accumulation in the SeD rat liver, kidney, and spleen [9], while another paper suggested age dependent differences of Fe contents in the SeD mouse liver [12]. Such ambiguities are probably due to variations of redox responses in given animals. The conclusions regarding the effects of Fe contents on oxidative stress depended on the experiment. In addition, the variation of Fe contents in the SeD rat liver may have a relationship with cytochrome P-450 activity as a redox response because the variation pattern Fe contents in the liver and P-450 activity of SeD rats was similar.

Fe contents increased with age in normal rat organs, but not showed particular tendency in Se-deficient rat. Fe content in the liver of younger Se-deficient rat looks fluctuating, but synchronizing with their P-450 activity. Our previous paper showed Fe contents increased in microsome fraction of Se-deficient rat liver [1,17]. This fluctuation of Fe contents probably is due to P-450 induction responding oxidative stress. Fe contents in 50 week old Se-deficient rat organs were, however, relatively low. That may be due to weak stress at this age, and the mechanisms of Fe accumulation in Se-deficient rat may be different from normal one.

The torula yeast based SeD diet is an artificially assembled diet consisting of several ingredients and nutrients [18]. The main minerals and vitamins in the diet are added at the recommended levels (Catalog of Oriental Yeast Co., LTD, Tokyo, Japan); however, unknown nutritional factors, for example inorganic cobalt (Co), other minor bio-essential elements, and so on, are not in that category. The Co content in the SeD diet is markedly low compared to a normal diet (CE-2, CLEA Japan, Inc., Tokyo), even though vitamin B_12_ is added to the SeD diet at the recommended level [1,2]; therefore some nutritional aspects of the SeD rat model remain ambiguous. It could also be argued that the low nutritional status in the torula yeast based diet, rather than the loss of GSH-Px activity creates some biological effects.

Se participates in the active centers of several enzymes in a biological system. A result of Se-deficiency obtained here was made by several different ways of biological responses. Therefore, clear relation of iron and zinc contents responding Se-deficiency can not be estimated from results. An important observation in this paper is that Se-deficiency is not lethal stress and responses of such minerals were different by rat ages, which may give different motional activities. A combination of an additional stresses on a Se-deficient animal is, however, still in progress.

## 3. Experimental

### 3.1. Materials

The SeD diet was purchased from Oriental Yeast Co., Ltd (Tokyo, Japan). The Se content of this SeD diet was determined in our previous study as 0.017 mg/kg by INAA [2]. Deionized water prepared by the Milli-Q system was used for all experiments and for drinking water for rats.

### 3.2. Animals

Pregnant Wistar rats were fed the SeD diet (Oriental Yeast Co., Ltd.) and ultra-pure water on the 15th day of pregnancy. Newly born rats were kept with their own mother for four weeks. Following this time, young rats were weaned, and then fed the SeD diet and ultra-pure water until the experiments. Male rats were used for experiments at 4, 6, 8, 12, 16, 20, and 50 weeks of age in the SeD groups. Normal male Wistar rats of the same age as the SeD group were purchased and used as normal control groups. The purchased normal rats had been fed on CLEA CE-2 diet (CLEA Japan, Inc., Tokyo) consistently before and after purchase. The Se content of the CE-2 diet was reported in our previous paper as 0.86 mg/kg by INAA [2]. Animal experiments were carried out in compliance with the Guidelines for Animal Care and Use at Showa Pharmaceutical University (2001), and were approved by the Ethical Committee for Animal Care and Use of Showa Pharmaceutical University.

### 3.3. Sample Preparation for Instrumental Neutron Activation Analysis (INAA)

The rats were anesthetized with intraperitoneally injected pentobarbital sodium (Nembutal, Abbott Laboratories, North Chicago, IL, USA) (50 mg/kg b.w.). Whole blood was drawn from the abdominal aorta, and then the whole body was perfused with ice-cooled saline (0.9% NaCl). The liver, kidney, and spleen were then removed. The organs were placed between two pieces of filter paper to allow the perfusion solution to drain, after which each organ was weighed. The organs from 5–6 rats were mixed and homogenized with a 4-fold volume of ultra-pure water. The homogenized organs were kept at -30 °C until the homogenate froze. The frozen samples were lyophilized and ground into powder. An aliquot (approximately 100 mg) of the powdered sample was weighed precisely and sealed in a quartz tube. The Standard Reference Material 1577b (bovine liver) obtained from the National Institute of Standards and Technology (Bethesda, MD, USA) was used to quantify the inorganic elements. Three tubes were prepared for each sample and subjected to thermal neutron irradiation in order to test the precision for analyses.

### 3.4. Neutron activation and data analysis

Neutron irradiation was carried out for 1 h at D-pipe (flux of thermal neutron = 4.3 × 10^13^ n/cm^2^s) in a JRR-4 nuclear reactor at the Japan Atomic Energy Research Institute. The irradiated samples were allowed to stand for at least for one week after irradiation. The γ-ray spectra of irradiated samples were measured with a high-purity germanium semiconductor detector equipped with a multichannel analyzer (Seiko EG&G Co., Ltd.). ^75^Se, ^59^Fe, and ^65^Zn were analyzed by photopeaks at 264.6, 1098.6, and 1115.4 keV, respectively [1,2]. The standard reference material 1577 (bovine liver) obtained from the National Bureau of Standards (Gaithersburg, MD) was used to quantify contents of elements in rat organ samples. The detection limit for each element was varied by experimental conditions, such as volume of sample, contents of other disturbance in the sample, energy of irradiation, and/or timing of measurement, but those were roughly estimated to be 4.1 mg/kg for Fe, 0.1 mg/kg for Zn, and 0.03 mg/kg for Se with this experimental condition.

### 3.5. Measurements of liver glutathione peroxidase (GSH-Px) activity

Five rats from each group were fasted for 24 hrs before the experiment and sacrificed by decapitation. The liver was perfused with ice-cold physiological saline (0.9% NaCl) until the blood was sufficiently removed. The liver was removed and homogenized with a 4-fold volume of 1.15% KCl in ice. GSH-Px activity in the liver homogenate of the current study was measured based on the method described by Paglia and Valentine [8]. 200 μL of PBS (pH 7.4), 50 μL 20 mM NaN_3_, 50 μL of 40 mM glutathione, 50 μL of 20 U/mL glutathione reductase, and 50 μL of 4 mM NADPH were added in a microtube. A portion (500 μL) of the diluted liver homogenate was added to the reaction mixture. The reaction was started by adding 100 μL of 1.0 mM H_2_O_2_. The time course of absorption at 340 nm (NADPH) of the reaction mixture was measured. GSH-Px activity was calculated from the slope of the plot of absorption with time. The consumption of 1 μmol NADPH per minute was converted as 1 U of GSH-Px activity. GSH-Px activity was standardized with protein concentrations and expressed as U/mg protein. The protein concentration of the liver homogenate was measured using a Bio-Rad Protein Assay Kit (Bio-Rad Laboratories, Hercules, CA).

### 3.6. Cytochrome P-450 contents in the liver microsome

Livers were homogenized with a 4-fold volume of 1.15% KCl in ice. After pre-centrifugation (9,000×g for 15 min, twice) to remove nuclear and mitochondrial fractions, a microsomal fraction was separated by ultra-centrifugation (100,000×g for 60 min, twice) of the supernatant. The microsomal fraction was suspended in a 2-fold volume of PBS (pH 7.4) and kept at -80 °C until the experiments. Cytochrome P-450 activity in the microsome was measured according to a method described by Omura and Sato [9]. A few milligrams of solid sodium dithionite (Na_2_S_2_O_4_) were added to the microsomal suspension, which was divided equally between two 1-cm square cuvettes, and the difference spectrum was recorded in the presence of CO. The content of P-450 was determined with a molar extinction difference of 91 cm^-1^mM^-1^. The cytochrome P-450 contents were standardized with protein concentrations and expressed as nmol/mg protein. The protein concentration of the microsomal suspension was measured using a Bio-Rad Protein Assay Kit (Bio-Rad Laboratories, Hercules, CA).

### 3.7. TBARS

The removed organs were homogenized with a 10-fold volume of saline (1.15% KCl). The TBARS levels of the homogenates were determined according to the method of Ohkawa *et al*. [10]. 200 μL of the homogenate, 200 μL of 8.1% SDS, 1.5 mL of 20% acetic acid, and 1.5 mL of 0.8% thiobarbituric acid was mixed and heated in boiled water for 60 min. After cooling, 1.0 mL of distilled water and 5.0 mL of *n*-butanol:pyridine (15:1) mixture were added and shaken. After centrifugation at 4000 rpm for 10 min, absorbance of the organic layer was measured at 535 nm. TBARS levels were calculated using 1,1,3,3-tetramethoxypropane as an external standard and expressed as nmol/g tissue.

### 3.8. Statistical test

Statistical differences were estimated with either Student’s or Welch’s T-test. A suitable test was automatically selected according to the variance of the data. Grades of significance were estimated by p < 0.05, p < 0.01, and p < 0.001.

## 4. Conclusions

In this paper, the growth curves of the body, liver, kidney, and spleen weight were compared between SeD and normal rats. The dependence of Se, Fe, and Zn contents in the liver, kidney, and spleen on the age of rats was also compared between SeD and normal rats. The amount of redox-relating minerals, such as Fe and Zn, in SeD rat organs is depending on the age. In conclusion, Se-deficiency is not lethal stress and responses of such minerals can be changeable by rat ages.
